# Experimental and Numerical Evaluation on the Performance of Perfobond Leiste Shear Connectors in Steel–SFRCC Composite Beams

**DOI:** 10.3390/ma15207237

**Published:** 2022-10-17

**Authors:** Kai Peng, Laijun Liu, Fangwen Wu, Song Lei, Jincheng Cao, Xiangyan Fan, Xuan Wang

**Affiliations:** 1 School of Highway, Chang’an University, Xi’an 710064, China; 2 CCCC First Highway Consultants Co., Ltd., Xi’an 710068, China

**Keywords:** Perfobond Leiste shear connector, steel fiber-reinforced cementitious composite, push-out tests, FE analyses, shear capacity equation

## Abstract

The difference between the shear performances of Perfobond Leiste (PBL) shear connectors embedded in steel fiber-reinforced cementitious composite (SFRCC) structure and normal strength concrete (NC) structure was investigated by push-out tests and finite element (FE) simulations. Push-out tests were carried out on nine steel-SFRCC specimens and nine steel-NC specimens. The mechanical behavior of the PBL shear connector was examined according to the failure modes, load-slip curves, and strain distribution laws of the push-out specimens. Experimental results revealed that the extension of cracks in SFRCC was hindered by steel fibers, and the number and width of cracks in SFRCC were smaller than those in NC. The failure mode of the steel-SFRCC specimens and the single-hole steel-NC specimens was the shear failure of the penetrating reinforcement, whereas that of the multi-hole NC specimens was concrete slab cracking. The ultimate shear bearing capacity of PBL shear connectors in the steel-SFRCC specimens was 47.8% greater than that in the steel-NC specimens. Furthermore, an FE model verified by the test results was established to conduct parametric analyses. It was found that the hole diameter and thickness of the steel plate and the yield strength of the penetrating rebar greatly affected the shear bearing capacity of PBL shear connectors. Finally, based on the experimental and FE simulation results, an expression for calculating the ultimate shear bearing capacity of PBL shear connectors in the steel-SFRCC composite structure was developed by considering the bearing effects of concrete dowels, penetrating rebars, and end parts.

## 1. Introduction

In modern bridge constructions, steel–concrete composite structures have been developed based on the reliability and economy of steel and concrete. Generally, steel girders and concrete slabs are connected by shear connectors. Under an applied load, concrete slabs become compressed and steel beams become elongated [1,2,3]. In composite beam bridges, shear connectors are mainly adopted to counteract the slippage and separation between steel beams and concrete slabs [4,5]. Perfobond Leiste (PBL) shear connectors are widely applied in large-span steel–concrete composite bridges due to their high stiffness in the elastic phase and good ductility in the plastic phase [6]. Several factors affect the mechanical behavior of PBL shear connectors, and among them, concrete properties play a key role [7]. Steel fiber-reinforced cementitious composites (SFRCC) are prepared using cement mortar as the base material and various discontinuous short fibers as used as reinforcing materials [8,9]. In recent years, SFRCC is greatly used in steel–concrete composite structures due to its high toughness and durability. The combination of PBL shear connectors and SFRCC can significantly enhance the mechanical behavior of steel–concrete composite beams.

PBL shear connectors are not single force-bearing components; thus, their stress mechanism is complicated. Numerical simulations and experimental investigations on the material properties and component sizes of PBL connectors have been conducted. Candido et al. [10] performed a push-out test on PBL shear connectors without penetrating rebars and found that the failure of PBL connectors occurred due to the shear failure of concrete dowels. Hu [11] demonstrated that the ultimate shear capacities of PBL shear keys increased with the increase in the number of holes. Nishiumi et al. [12] investigated the effects of hole diameter, concrete compressive strength, and penetrating steel rebars on the ultimate bearing capacities of PBL shear connectors and asserted the role of the penetrating reinforcement as a lateral constraint. Zhang et al. [13,14] considered the similarities and differences of bearing capacity formulas in the ductile and brittle failures of the penetrating reinforcement and studied the force transmission mechanism of PBL shear connectors. Ahn et al. [15] considered the chain effect between holes and the influence of the spacing between each row of shear keys and proposed a reasonable spacing between rows of shear keys and holes on flange plates. Zheng and Liu [16,17] proposed a calculation method for the initial shear stiffness of PBL shear keys based on the “elastic foundation beam theory” and examined the effect of rib spacing on shear stiffness. Zhan et al. [18] analyzed the behavior of PBL connectors under external pressure to simulate the performance of the connectors in tower columns subjected to long-term large lateral pressure.

Steel–NC composite beams with PBL connectors have different degrees of concrete voids and cracking, which yield adverse effects on structural stress and durability [19,20]. High-performance concretes have excellent mechanical properties. Reactive powder concrete (RPC) and ultra-high performance concrete (UHPC) are generally applied to steel–concrete joint sections to ensure pouring quality and effectively improve mechanical performance and structural durability [21]. Zhang long [22] used NC and RPC as pouring materials, respectively, to perform an experimental study on the shear bearing capacities of 20 stud shear key and PBL shear connector specimens and reported that all specimens acted as shear transmission members. He et al. [23] used UHPC as a grouting material and reported that UHPC greatly improved the shear capacities of PBL connectors by reducing the bond coefficient between steel and concrete and delaying cracking time. Although RPC and UHPC can effectively enhance the mechanical behavior and ultimate shear capacity of steel–concrete composite structures, RPC requires active materials and steel fibers. In addition, autoclave curing or thermal curing is generally adopted for RPC to ensure high strength and good durability, resulting in a high manufacturing cost [24]. Furthermore, UHPC has a relatively high preparation cost, a relatively complex design, high cementitious material consumption, and a low water–cement ratio, which leads to large shrinkage and self-shrinkage [25].

SFRCC is a multiphase heterogeneous composite material consisting of fine aggregates, coarse aggregates, cement, and randomly distributed steel fibers. Steel fibers can refine the microstructure of concrete and reduce internal defects. Tiberti et al. [26] explored the ability of fibers in controlling cracks by discussing more than ninety tension tests on reinforced concrete prisms. More et al. [27] investigated the mechanical and durability qualities of fiber-reinforced concrete (FRC) experimentally with the optimum fraction, and a constitutive model for the analysis of reinforced concrete structures was modified to improve its computational efficiency [28]. Tamrazyan et al. [29] studied the solution of the analysis of the stress–strain state of FRC structures under emergency dynamic effects. Some numerical models were presented and verified as able to represent the behavior of cracked steel fiber reinforced concrete (SFRC) [30,31]. The addition of steel fibers can significantly limit the shrinkage and cracking of concrete, increase the toughness and tensile yield strength of concrete, and improve the plastic shrinkage performance, impermeability, frost resistance, and dynamic load performances (impact resistance and fatigue) of concrete [32,33,34,35]. In addition, SFRCC can be easily prepared and it is suitable for a variety of working conditions. Moreover, SFRCC costs less than UHPC. Hence, SFRCC has wide application prospects in steel–concrete composite structures. However, existing experimental research on the static behavior of PBL connectors mainly concentrates on the use of normal concrete or high-performance concrete. Moreover, existing experimental investigations generally use a few test pieces and have incomplete considerations of influencing factors. Therefore, theoretical and experimental research on PBL connectors needs to be further deepened.

To investigate the failure mechanism of PBL shear connectors in SFRCC and determine the calculation formula of shear bearing capacity, nine steel–SFRCC composite structures and nine steel–NC composite structures with PBL shear connectors were designed in the present work. The effects of penetrating rebar diameter, the number of steel plate openings, and concrete type on the ultimate shear bearing capacity, shear stiffness, failure modes, load-slip curves, and load–strain curves of the specimens were discussed, and the main differences between the stress characteristics of the steel–SFRCC and steel–NC structures were revealed. Moreover, the effects of SFRCC strength, steel plate opening diameter and thickness, and penetrating reinforcement yield strength on the ultimate shear capacity of PBL shear keys were systematically analyzed by a FE model (verified by test results). Finally, an expression for calculating the ultimate shear bearing capacities of PBL shear connectors in the steel–SFRCC composite structure was developed.

## 2. Experimental Program

### 2.1. Specimens Design

**Figure 1:**
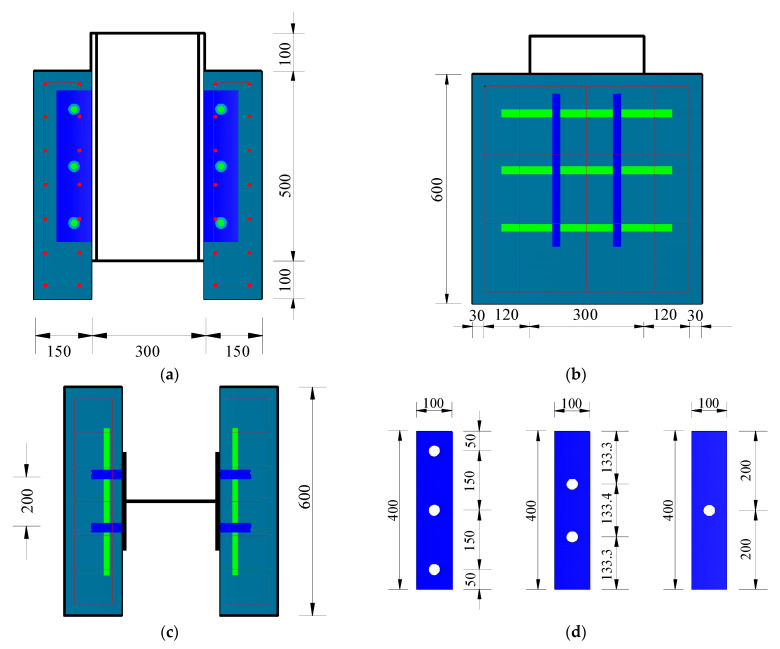
Specimen dimensions: (**a**) front view; (**b**) side view; (**c**) plan view; and (**d**) perforated steel plate (unit: mm).

According to Eurocode 4 [36], nine steel–NC and nine steel–SFRCC push-out specimens were prepared to examine the mechanical properties of PBL connectors (Figure 1). PBL steel plates of 10 mm thickness were made of Q345D bridge steel provided by HaoXing Co., Ltd (Yancheng, China). Single-hole, double-hole, and three-hole steel plates were used, and the diameter of each hole was 40 mm. The dimension of H-shaped steel beams was 300 mm × 300 mm × 10 mm × 15 mm, and HRB400 steel bars provided by HaoXing of different diameters (12 mm, 16 mm, and 20 mm) and a length of 450 mm were used as penetrating rebars. To guarantee the structural reliability of the specimens during push-out tests, j506 welding rods provided by HaoXing were employed to connect the PBL steel plates to the section steel beams, and stirrups adopted HRB400 reinforcement with a diameter of 8 mm crossing the opening steel plates were cut off. The specimen numbering format was “N/S-12/16/20-1/2/3,” where N represents NC, S represents SFRCC, 12/16/20 represents the penetrating rebar diameter, and 1/2/3 represents the number of steel plate holes.

### 2.2. Specimen Fabrication

**Figure 2:**
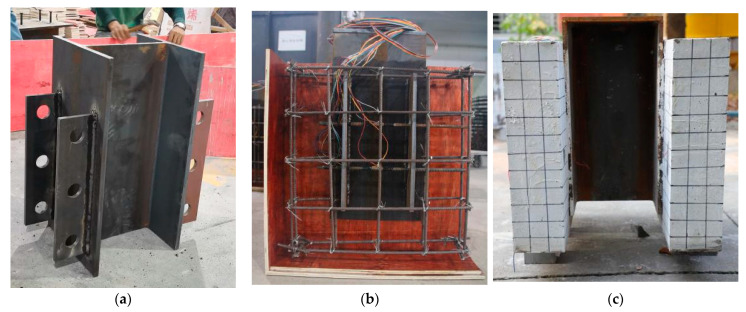
Fabrication of specimens: (**a**) PBL production; (**b**) template assembling; and (**c**) completed specimen.

The fabrication process of a test specimen is displayed in Figure 2. First, an H-shaped steel beam was processed, polished, and derusted before welding. Prior to perforated steel plate welding, the PBL position on the surface of the profile steel surface was accurately set out, and the girth welding method was adopted to improve welding quality. A reinforcement mesh was assembled with the steel structure, and the interior of the frame and the profile steel plate surface were painted with oil to facilitate demolding and reduce the bonding effect between the concrete slabs and the profiled steel plates. Finally, concrete was poured into the steel frame. It should be noted that the concrete vibrated continuously during pouring. After removing the concrete structure from the mold, it was water cured.

### 2.3. Material Properties

**Figure 3:**
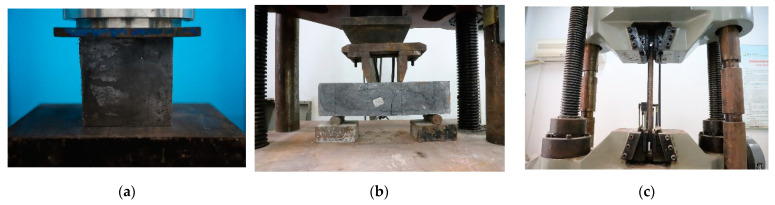
Measurement of mechanical properties: (**a**) concrete compression test; (**b**) concrete bending test; and (**c**) rebar tensile test.

Commercial concrete was used for NC and SFRCC was produced by the Sino-German New Asia (Xinmi, China). Table 1 displays the mixing ratios of NC and SFRCC. The composition of SFRCC contains silicon powder and hooked-end steel fibers with a length of 30 mm and a diameter of 0.5 mm, but not in NC. Three groups of 100 × 100 × 100 mm^3^ cubic specimens and 150 × 150 × 550 mm^3^ prism specimens were fabricated to test the compressive and tensile strengths of concretes. The tensile strength of HRB400 steel bars was measured with a universal testing machine produced by Hailian (Dezhou, China), (Figure 3) and the mechanical parameters of Q345D steel were provided by the manufacturer. The mechanical performances of the concretes and steel are presented in Table 2.

### 2.4. Test Setup

**Figure 4:**
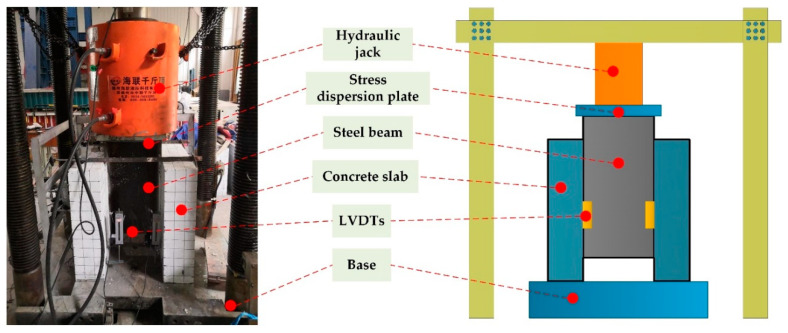
Push-out test device.

The push-out test device produced by Hailian used in this experiment is displayed in Figure 4. Each specimen was loaded by a 500 t press and left–right symmetrical displacement gauge linear variable displacement transducers (LVDTs) were set at the bottom of the H-shaped steel beam and the concrete slab to measure the relative slip at their interface. According to Eurocode 4 [36], each specimen was preloaded with a load of 20% of its theoretical ultimate bearing capacity. It should be noted that for each sample, the push-out test was repeated three times. In the initial loading stage, the specimens were pressed step by step (10 kN load per stage). When the specimens entered the plastic stage, the loading rate was reduced to 5 kN/min to record their failure processes in detail.

## 3. Experimental Results

### 3.1. Failure Modes

**Figure 5:**
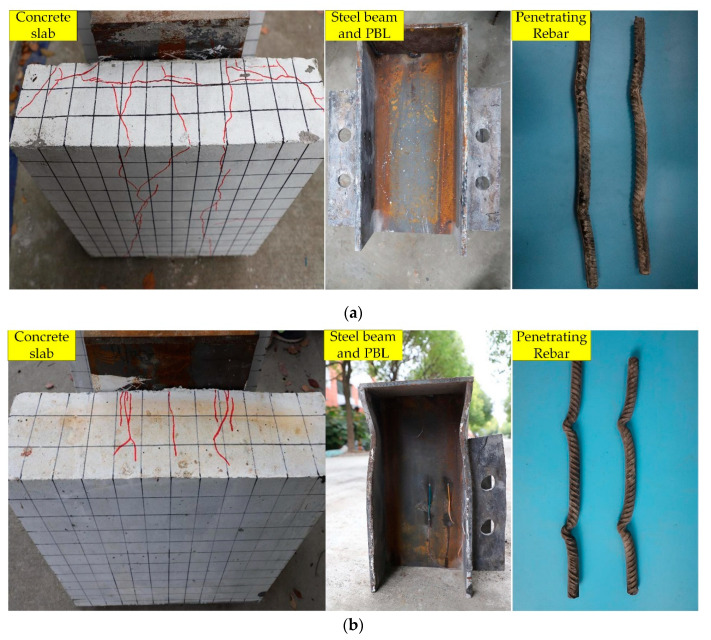
Failure modes of PBL shear connectors: (**a**) N-16-2 and (**b**) S-16-2.

The typical failure modes of the steel–NC and steel–SFRCC structures are displayed in Figure 5. In the steel–SFRCC composite structure, very few microcracks appeared on the top and inner surfaces of the concrete; however, no cracks were noticed on the outer surface. In contrast, numerous cracks existed on the top, outer, and inner surfaces of the concrete in the steel–NC structure. In the steel–SFRCC structure, the cracking load was large, crack development speed was slow, crack width was small (0.5–1 mm), crack continuity was strong, and cracks were relatively regular, whereas cracks in the steel–NC structure were irregular. The upper part of the steel plate in the steel–SFRCC structure buckled, and the top of the shear connector opening was squeezed by the penetrating reinforcement, resulting in obvious distortion and deformation, whereas the H-shaped steel beam and the steel plate opening in the steel–NC composite structure did not deform significantly. The deformation of the penetrating reinforcement in the steel–NC composite structure was relatively small and resulted in a shear failure. In the steel–SFRCC composite structure and the single-hole steel–NC specimen, the bending deformation of the penetrating reinforcement was large, necking occurred near the fracture, and the failure mode of the penetrating reinforcements was bending shearing.

The failure modes of the SFRCC specimens were different from those of the NC specimens. The failure of the single-hole steel–NC specimen happened due to the bending shearing of the penetrating reinforcement, whereas the multi-hole steel–NC specimen fractured because of the cracking of the concrete slabs. In contrast, the failure of the SFRCC specimens could be attributed to the bending shearing of the penetrating reinforcement. During the test, the shear force was transmitted between the PBL plates and the concretes, generating local stress in the composite structure. The local concrete was subjected to large pressure and splitting force. As SFRCC has high strength and can bear large loads and the bridging effect of steel fibers can effectively limit the expansion of cracks, the failure of PBL connectors in steel–SFRCC occurred due to the bending shearing of the penetrating reinforcement. Due to the insufficient strength of NC, the concrete at the bottom of the PBL steel plate was crushed by excessive local pressure during the test, generating a large number of cracks in the concrete; thus, the concrete plate cracked and could not create effective constraints on PBL shear connectors. Therefore, shear connectors with high bearing capacity should be used to achieve the high-strength performance of SFRCC.

### 3.2. Load–Slip Curves

**Figure 6:**
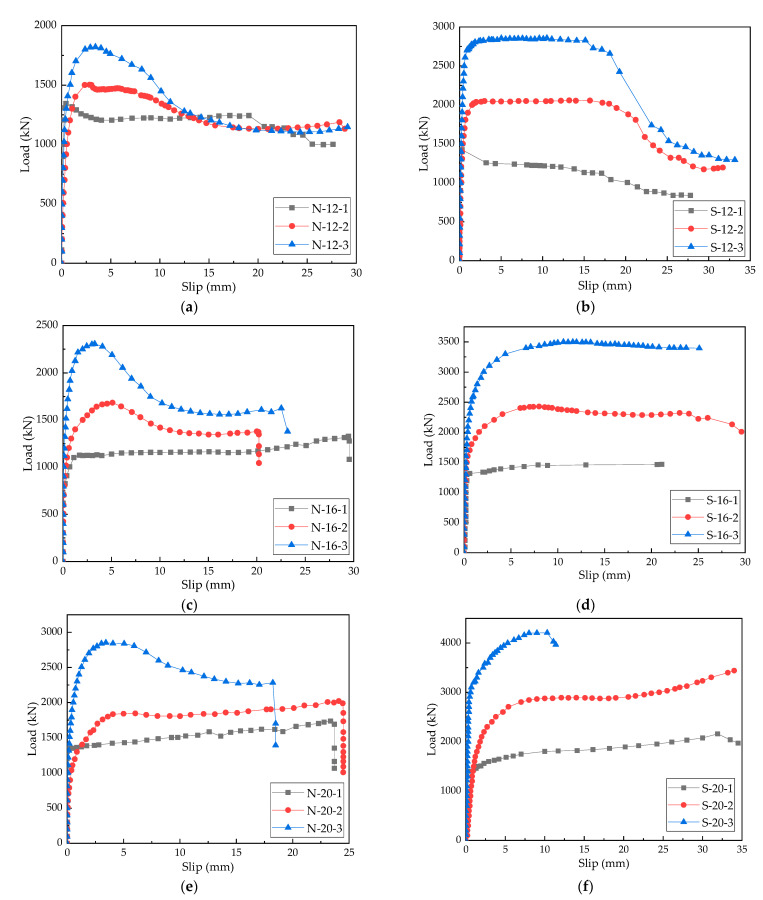
Load–slip curves of specimens: (**a**) N-12; (**b**) S-12; (**c**) N-16; (**d**) S-16; (**e**) N-20; and (**f**) S-20.

The load–slip curves of the push-out test specimens are exhibited in Figure 6. It is noticeable that the load–slip curves went through four stages: elastic stage, nonlinear stage, strengthening stage, and failure stage. In the elastic stage, the load–slip curves were oblique straight lines and the load growth rate was proportional to the relative slip value, indicating that PBL shear keys were in the elastic deformation stage and the penetrating reinforcement of the shear keys and the concrete worked together to resist shearing. At this time, the shear connectors did not enter the normal stress state. In the nonlinear stage, the load value reaches 70–80% of the ultimate shear capacity and the slope of the load–slip curve gradually decreased. The load–slip curves changed from oblique lines to convex curves, indicating that both elastic and plastic deformations occurred in this stage. Concrete dowels in the PBL specimens and the surrounding concrete entered the plastic deformation stage, and the embedding effect on shear keys decreased, increasing the relative slip growth rate. As the test continued, the penetrating reinforcement and the perforated steel plate gradually yielded, and cracks appeared in the concrete slabs, further accelerating the growth of relative slip. In the strengthening stage, the slope of the load–slip curves further decreased until they became nearly parallel to the X-axis, indicating that the deformation of the specimens further increased in this stage, whereas the load change was small. Concrete dowels were crushed, the penetrating reinforcement was greatly bent, and the perforated steel plates began to deform. The relative slippage between the steel plate and the concrete slabs was visible to the naked eye, and some NC slabs were severely cracked. The load in the failure stage reached the maximum value, the push-out specimens were noticeably deformed, and internal shear connectors and concrete slabs were irreversibly damaged. With the further progress of the test, the load did not increase; however, the relative slip value still increased rapidly. When the deformation exceeded the ultimate deformation of shear connectors or concrete slabs, the specimens were seriously damaged and could not bear the load.

When the failure occurred, the ultimate slip amount of the steel–SFRCC composite structure was larger than that of the steel–NC composite structure. This happened because due to the presence of steel fibers in the steel–SFRCC composite structure, cracks were not developed rapidly after their appearance; thus, the structure continued to bear the load. However, the failure of the steel–NC composite structure was faster due to the second-order effect of cracks.

Eurocode 4 [36] stipulates that $p_{u}$ represents the ultimate load of the test piece; the characteristic value of bearing capacity ($p_{Rk}$) is 90% of the ultimate load ($p_{u}$), and Equation (1) is defined to calculate the design value ($P_{Rd}$) of bearing capacity:

(1) $$P_{Rd}=\frac{f_{u}}{f_{ut}}\frac{P_{Rk}}{\gamma_{v}}\leq \frac{P_{Rk}}{\gamma_{v}}$$

where $f_{u}$ is the design strength of the material; $f_{ut}$ is the actual ultimate strength of the material; and $\gamma_{v}$ is the safety factor, taking 1.25. The displacement ductility coefficient (D) refers to the ratio of the slip amount ($\delta_{u}$) of the test piece under the ultimate load to the slip amount ($\delta_{Rd}$) corresponding to the design load. Its expression is shown in Equation (2):

(2) $$D=\frac{\delta_{u}}{\delta_{Rd}}$$

The secant slope corresponding to the interfacial slippage of 0.5 mm was taken as the shear stiffness value (K) [37]. The calculation results of the static performances of different specimens are listed in Table 3.

### 3.3. Load–Strain Curves

#### 3.3.1. Concrete Slabs

**Figure 7:**
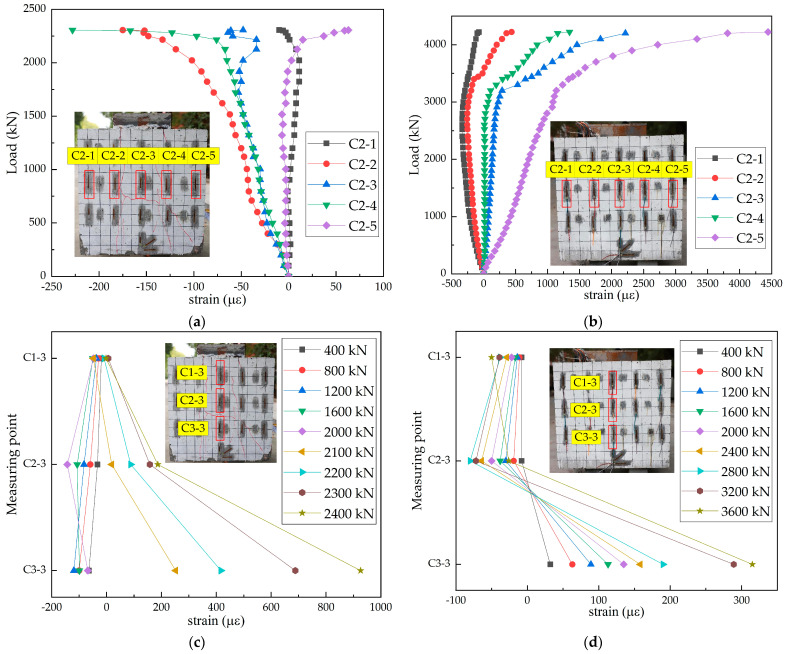
Load–strain curves of concrete: (**a**) N-20-3; (**b**) S-20-3; (**c**) N-20-3; and (**d**) S-20-3.

Taking the three-hole specimen as an example, the load–strain curves of the concrete slabs in the steel–SFRCC and steel–NC composite structures were plotted (Figure 7). In the initial loading stage, the strain of the concrete slab in both structures was small and increased almost linearly, and generally conformed to the assumption of plane section, indicating that the structures were in the linear stage at this time. When the load was approximately 0.86 times the ultimate load, the strain increased rapidly, leading to a rapid crack development in the concrete slabs. When the ultimate shear capacity was achieved, the strain of the concrete slabs in the steel–SFRCC composite structure continued to increase. It happened because after the appearance of cracks, steel fibers in SFRCC greatly impeded the propagation of cracks in the concrete slab; thus, the structure continued to bear the load. However, after the steel–NC composite structure reached the ultimate load, cracks were developed in the concrete slabs rapidly, resulting in a rapid failure of the structures and a sharp decrease in bearing capacity.

#### 3.3.2. H-Shape Steel Beams

**Figure 8:**
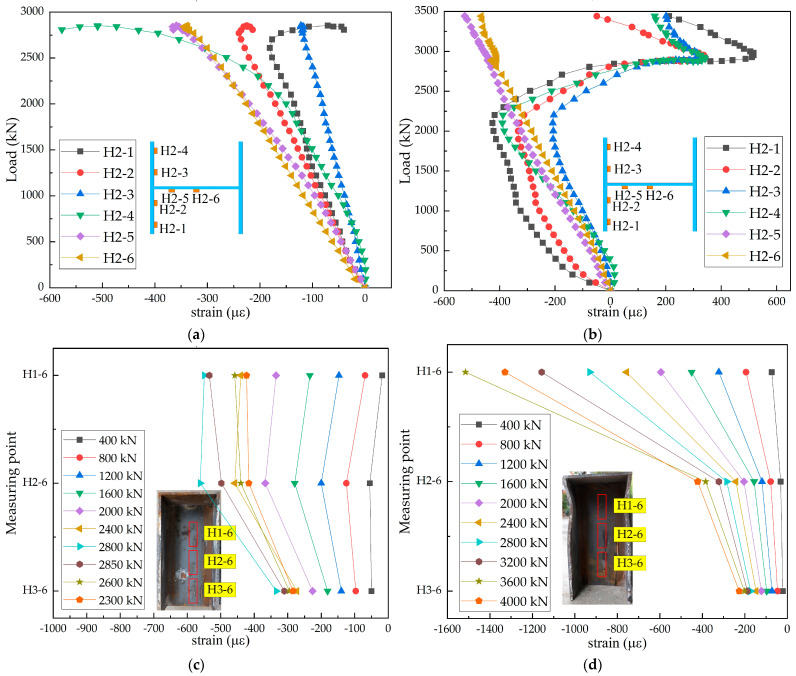
Load–strain curves of H-shaped steel beams: (**a**) N-20-3; (**b**) S-20-3; (**c**) N-20-3; and (**d**) S-20-3.

The load–strain curves of H-shaped steel beams in the steel–SFRCC and steel–NC composite structures are displayed in Figure 8. The strain in the initial loading phase was small and increased almost linearly. When the load was approximately 0.86 times the ultimate load, strains at measuring points 1, 2, 3, and 4 of the H-beam in the steel–SFRCC composite structure suddenly inversely increased, and buckling deformation occurred at the contact section between the PBL steel plates and the H-beam; whereas the H-shaped steel beam in the steel–NC composite structure experienced no deformation. The existence of a large number of steel fibers, which had good tensile and shear properties, in SFRCC led to the buckling deformation of the H-shaped steel beams. The upper area of the H-shaped steel beams in both structures was subjected to a large stress, whereas the lower area was subjected to smaller stress.

#### 3.3.3. Penetrating Rebar

**Figure 9:**
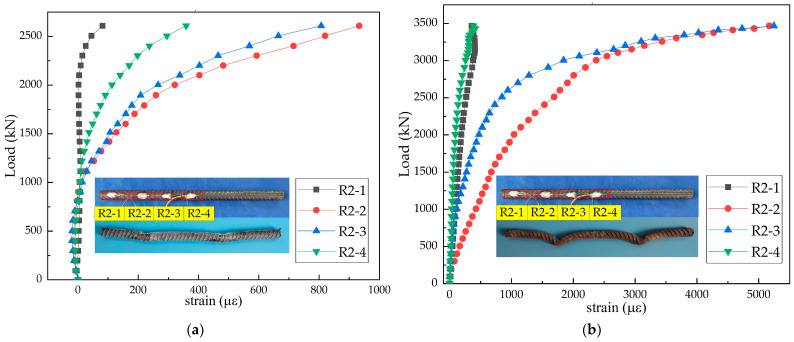
Load–strain curves of penetrating rebar: (**a**) N-20-3 and (**b**) S-16-3.

Strain gauges in the penetrating reinforcement were arranged along the axial direction of the reinforcement to monitor the stress state when the penetrating reinforcement was subjected to axial deformation or bending deformation. It is observable from Figure 9 that in the initial loading phase the strain of the penetrating reinforcement was small, implying that the penetrating reinforcement remained straight at this time and concrete dowels bore the load. As the test progressed, concrete dowels gradually failed, and penetrating rebars in holes began to bend and shear. When concrete dowels were sheared, the load induced by the dowels was transferred to the penetrating reinforcement. The stress of the penetrating reinforcement was similar to that of an elastic foundation beam supported on concrete blocks at both ends, and the penetrating reinforcement bore the concentrated load on the perforated steel plates; thus, the bending strain at the loading point continued to increase. In both NC and SFRCC, strains at measuring points 2 and 3 near the perforated steel plate on the penetrating reinforcement were greater than those at measuring points 1 and 4, indicating that the closer to the perforated steel plates, the greater the strain on the penetrating steel bar.

#### 3.3.4. Perforated Steel Plate

**Figure 10:**
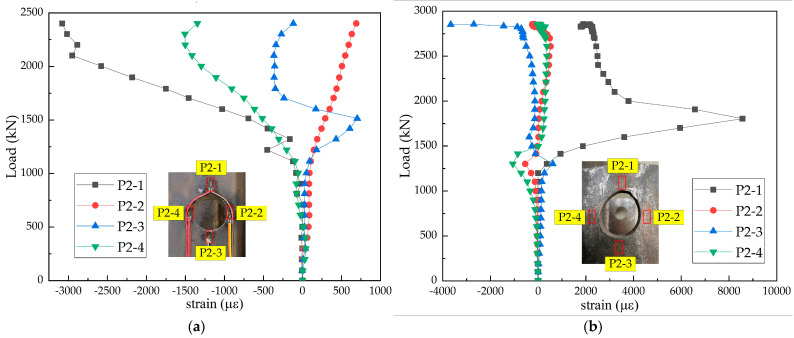
Load–strain curves of perforated steel plate: (**a**) N-16-3 and (**b**) S-16-3.

When PBL shear connectors were stressed, due to the low strength of concrete, concrete dowels were easily crushed and plastic flow occurred in the core stress area of the connectors; thus, the penetrating reinforcement was squeezed to the edge of the perforated steel plates and came in contact with the steel plate. When the penetration reinforcement diameter was small, it was cut off by the perforated steel plates. When the perforated steel plate was thin, it was squeezed and deformed by the penetrating reinforcement. The strain values at steel plate openings in the two types of composite structures are presented in Figure 10. Significant differences were noticed between PBL steel plate openings in the steel–SFRCC and steel–NC composite structures. The PBL steel plate opening in the steel–SFRCC composite structure was squeezed by the penetrating reinforcement, and the concrete strength at the end of the PBL plate was high, resulting in obvious deformation of the P2-1 measuring point at the top of the PBL plate opening and the penetrating steel bar. However, in the steel–NC composite structure, the concrete at the bottom of the PBL steel plate was crushed by excessive local pressure, forming a large number of cracks in the concrete slabs. Finally, the cracking of the concrete slab failed to create effective constraints on PBL shear connectors, and the steel plate did not produce obvious deformation.

## 4. FE Analyses

### 4.1. FE Model Definition

**Figure 11:**
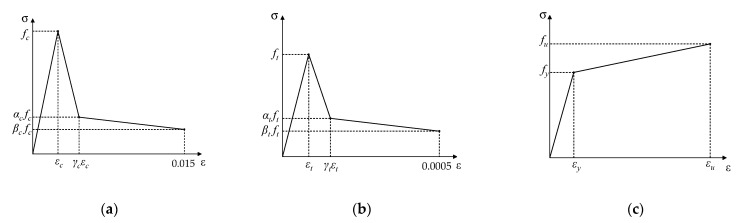
Constitutive laws of materials: (**a**) compressive behavior model of SFRCC; (**b**) tensile behavior model of SFRCC; and (**c**) bifold line model of steel.

The FE model was built by ABAQUS 6.14-4 and a half model was developed according to the geometrical symmetry of the push-out test specimens. The H-shaped steel beam, the concrete, the penetrating reinforcement, and the perforated steel plate were simulated by the solid element, whereas the truss element was applied to simulate the embedded reinforcement. The three-line constitutive model proposed by Shi [38] was adopted for the SFRCC, and the bifold line model was employed for the H-shaped steel plate and the penetrating rebar (Figure 11). The ultimate strain is selected as 0.015 for compression and 0.0005 for tension, where $f_{c}$ and $f_{t}$ are respectively the compressive strength and tensile strength of SFRCC, and $\varepsilon_{c}$ and $\varepsilon_{t}$ are respectively the compressive strain and tensile strain of SFRCC. The coefficients α and β are used to normalize the post-cracking stress to the peak stress, while the coefficient γ is defined as the ratio between the strain at the end point of the descending branch and the strain at the peak load. $f_{y}$ and $f_{u}$ represent the yield strength and ultimate strength of steel, respectively, and $\varepsilon_{y}$ and $\varepsilon_{u}$ represent the yield stress and ultimate stress of steel, respectively.

**Figure 12:**
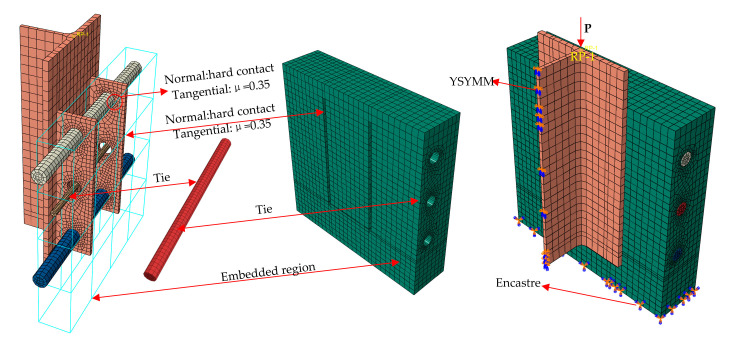
FE model.

To ensure that the model did not have sharp and deformed meshes during mesh generation, its different parts were cut several times and local seeds were laid out before mesh generation. Moreover, the irregular joint parts were divided into geometric components of regular shapes; especially, densified seeds were distributed around circular holes. The concrete dowels and the concrete were simulated by a “tie” contact. The perforated steel plates and the H-shaped steel plates were welded and considered a whole without any contact. The surface-to-surface contact was adopted between the penetrating reinforcement and the concrete dowels, hard contact was used in the normal direction, and a penalty function with a coefficient of 0.5 was adopted in the tangent direction. Hard contact was applied between the PBL shear connectors and the concrete in the normal direction, and a penalty function with a coefficient of 0.1 was used in the tangent direction. Hard contact was also used between the perforated steel plates and the concrete dowels in the normal direction, and a penalty function with a coefficient of 0.904 was adopted in the tangent direction. Embedded restraints were introduced between the stirrups and the concrete. A symmetrical constraint was utilized to the center of the steel beam and a vertical constraint with six degrees of freedom was used for the concrete base plate. In order to facilitate load application and result extraction, a reference point RP1 was established at the top of the specimen, a coupling constraint was adopted between RP1 and the top surface of the model, and displacement loads were applied at RP1 according to a smoothed magnitude function (Figure 12).

### 4.2. FE Model Validation

**Figure 13:**
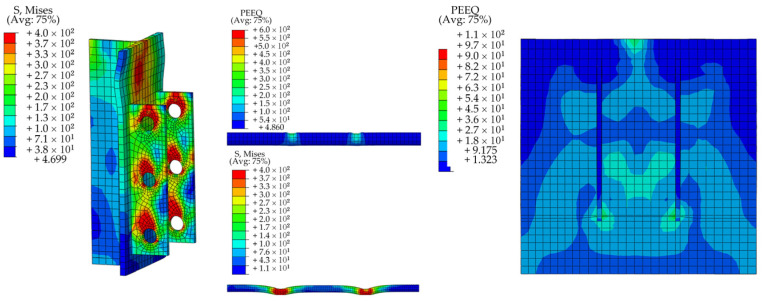
Stress contour of the FE model.

Figure 13 displays the stress contour of the FE model for the S-16-3 specimen. The upper part of the H-shaped steel beam experienced large stress and buckling deformation in the out-of-plane direction indicating that the top of the steel beam acted as a lateral limit to the SFRCC plate. Stress concentration occurred at the roof of the PBL steel plate opening and the top of the contact between the PBL plate and the steel beam. The contact section between the concrete dowels and the PBL plate was crushed, and the penetrating steel bars yielded at the stressed section. The stress level of the SFRCC plate was low, and stress concentration in the end region of the perforated plate was relatively large and gradually decreased radially around the concrete block.

**Figure 14:**
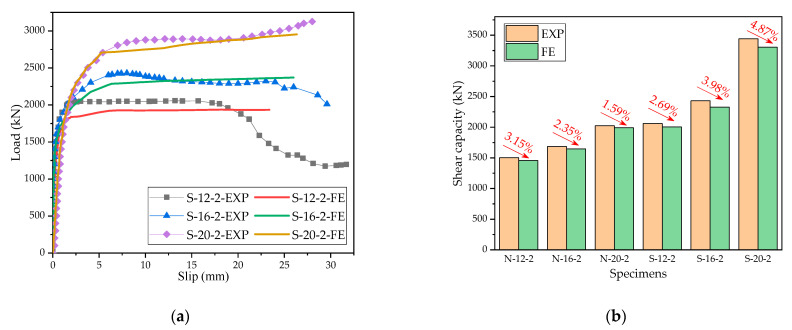
Comparison of experiment and FE results: (**a**) load–slip curves and (**b**) shear capacity.

A comparison between the experimental and simulated load–slip curves is presented in Figure 14a. It is noticeable that the FE models could well simulate the load–slip relationship of the specimens in the elastic stage, the nonlinear stage, and the strengthening stage and also successfully analyze ultimate shear capacities. A comparison between experimental and simulated ultimate shear bearing capacities is presented in Figure 14b. The error of the FE analysis was smaller compared with that of the push-out test. The minimum and maximum errors of the shear capacity obtained from the FE model and the push-out test were 1.59% and 4.23%, respectively (both within 5%), indicating that the numerical results are reliable. The ultimate shear capacity values obtained from the FE models were slightly smaller than those obtained from the push-out test because the concrete dowels of PBL shear keys failed before the penetrating reinforcement in the actual test. However, in the FE model, a great unbalanced force appeared in the nonlinear analysis after the failure of the concrete, making it difficult for nonlinear iterations to converse. When the number of iterations reached a certain limit, the program generated a nominal calculation result, which was very close to the real value. The FE model successfully simulated the load–slip laws in the elastic stage, the nonlinear stage, and the strengthening stage; however, it could not well simulate the load–slip relationship in the failure stage. It happened because the constitutive relationships of different materials were optimized during modeling, for example, the reinforcement adopted an ideal elastic–plastic model. Although the FE model could not simulate the load–slip relationship in the failure stage, it accurately analyzed the ultimate shear capacity. Therefore, the FE model can be adapted to carry out the parametric analyses of the shear capacities of PBL keys in the steel–SFRCC composite structure.

### 4.3. Influences of Different Parameters

**Figure 15:**
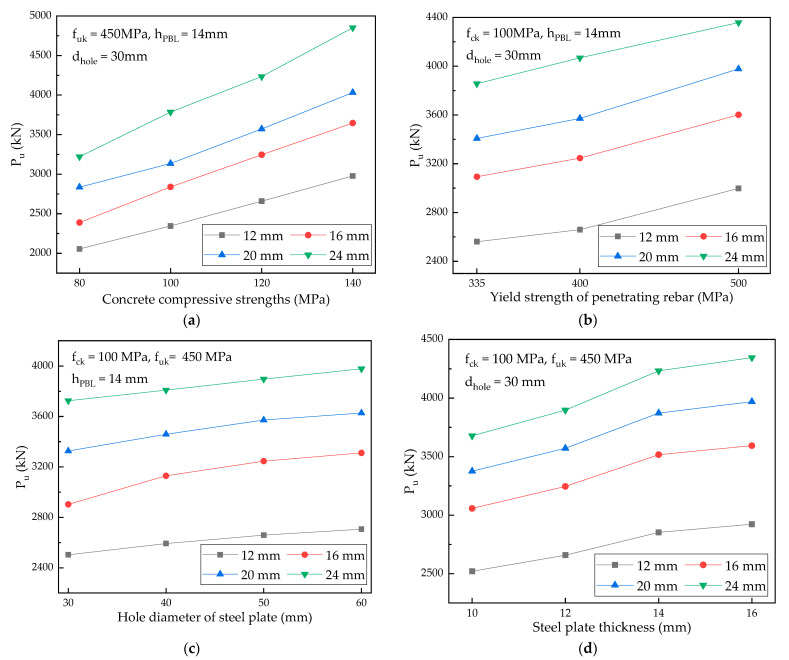
Parameter analyses: (**a**) concrete compressive strengths; (**b**) yield strength of penetrating rebar; (**c**) hole diameter of steel plate; and (**d**) steel plate thickness.

The parametric analysis was conducted based on the control variable method and the influences of different parameters on the shear bearing capacities of PBL shear connectors were examined by the FE model. Four different penetrating reinforcement diameters (12 mm, 16 mm, 20 mm, and 24 mm) were adopted for the parametric analyses (Figure 15).

(a) Concrete strength: The ultimate shear capacity of the PBL connector increased with the increase in concrete compressive strength; however, the increment range gradually became smaller. When the concrete compressive strength increased from 80 MPa to 140 MPa by 20 MPa increments, the ultimate shear capacities of PBL connectors increased by approximately 18.84%, 14.36%, and 12.32%. High-strength concrete enhances the shear capacities of concrete dowels and limits the extension of cracks; however, when the concrete strength increases to a certain value, the shear bearing capacities of connectors are determined by other parameters such as the penetrating rebar and steel plate.
(b) Yield strength of the penetrating reinforcement: When the yield strength of the penetrating reinforcement strength increased from 335 MPa to 400 MPa, the shear capacity of PBL connectors increased by approximately 4.96%. When the reinforcement strength increased from 400 MPa to 500 MPa, the shear capacity of PBL connectors increased by approximately 9.25%. When the area of the penetrating rebar was fixed, the strength increment of the penetrating steel bar improved the ultimate shear capacities of PBL connectors after the failure of concrete dowels; thereby, the overall shear capacity of the structure was enhanced.
(c) Steel plate hole diameter: The shear capacity of PBL connectors increased with the increase in the steel plate opening diameter; however, the increment was small (within 5%). The shear capacity of PBL connector was improved to a certain extent because the increase in the PBL opening diameter increased the shear resistance area of concrete dowels.
(d) Steel plate thickness: The shear capacities of the end-bearing PBL connectors increased with the increase in the perforated steel plate thickness. When the steel plate thickness increased from 10 mm to 16 mm by 2 mm increments, the shear capacities of PBL connectors increased by approximately 6.15%, 8.32%, and 2.79%. The steel plate thickness affected the compressive area of the concrete dowels and the penetrating reinforcement and directly bore the compressive force induced by the concrete under the perforated steel plate.

## 5. Theoretical Analysis

Numerous formulas have been established to calculate the ultimate shear capacities of PBL connectors in steel–NC and steel–UHPC composite structures. Table 4 summarizes the existing formulas for calculating the shear capacities of PBL connectors embedded in steel–concrete composite structures, and Table 5 compares the theoretical and experimental shear capacities of PBL shear connectors. It is notable that the proposed calculation formulas have a high degree of agreement with the calculation formula in the specification. However, they could not successfully calculate the ultimate shear capacities of the PBL connector in the steel–SFRCC composite structure. As the influence of steel fibers in the concrete was not considered, the calculation results of Equations (3)–(5) for the shear bearing capacities of PBL connectors in NC were relatively small, whereas the calculation result of Equation (6) in UHPC was relatively large. The material properties of SFRCC and traditional concrete are quite different; thus, it is necessary to propose a calculation formula for the shear-bearing capacities of PBL connectors in SFRCC.

The shear capacities of the PBL shear keys were mainly composed of three parts: the action of penetrating reinforcement ($P_{pr}$), the action of the concrete dowels ($P_{cd}$), and the bearing action of concrete under the steel plate ($P_{eb}$). The shear bearing capacities composition of PBL shear connectors in SFRCC were different from that of NC due to the “bridging” effect of steel fiber. In this paper, the “bridging” effect of steel fibers was considered in the contribution of the concrete dowels to the longitudinal shear capacities when calculating the bearing capacity. Therefore, the shear bearing capacity ($P_{u}$) of the PBL shear key in SFRCC can still be expressed by the above three parts:

(7) $$P_{u}=P_{pr}+P_{cd}+P_{eb}$$

The bearing capacity of the penetrating reinforcement was mainly dependent on its strength and was hardly affected by the concrete type and steel fibers. The penetrating reinforcement was subjected to the combined effect of bending moment and shear force during loading, and the shear strength of the reinforcement was related to its yield strength. In this analysis, the yield strength of the reinforcement ($f_{y}$) was taken as the material strength index, and the force of penetrating reinforcement ($P_{pr}$) in steel plate holes was calculated by introducing the coefficient $k_{1}$:

(8) $$P_{pr}=4n\cdot k_{1}\cdot A_{pr}\cdot f_{y}$$

where n is the number of holes in steel plate and $A_{pr}$ is the cross-sectional area of the penetrating reinforcement.

CECS code [41] recommended the formula of shear bearing capacity ($P_{cs}$) of SFRCC as follows:

(9) $$P_{cs}=\left(1+kV_{f}\frac{L_{f}}{\phi_{f}}\right)\cdot P_{c}$$

where k is the coefficient to be determined; $V_{f}$ is the volume fraction of steel fiber; $L_{f}$ is the length of steel fiber; $\phi_{f}$ is the diameter of steel fiber; and $P_{c}$ is the shear bearing capacity of non-steel fiber concrete.

The pin and bolt action of concrete dowels was mainly determined by their shear strength, this effect could be expressed by considering the reinforcement action of steel fibers on the shear resistance of concrete:

(10) $$P_{cd}=4n\cdot k_{2}\cdot A_{cd}\cdot f_{c}\cdot \left(1+kV_{f}\frac{L_{f}}{\phi_{f}}\right)$$

$k_{2}$ is the coefficient to be determined and $A_{cd}$ is the area of concrete dowels.

The bearing action of the concrete under the PBL plate was mainly determined by the end region of the perforated steel plates and the compressive strength of the concrete. Its calculation formula could be expressed as:

(11) $$P_{eb}=k_{3}\cdot A_{eb}\cdot f_{c}$$

where $k_{3}$ is the coefficient to be determined and $A_{eb}$ is the contact area between the steel beams and the concrete slabs.

According to the above analysis, a comprehensive regression calculation model with clear physical meaning could be proposed, where $k_{1}$, $k_{2}$, $k_{3}$, and k are the regression coefficient, which could be obtained by using SPSS for regression analysis. Based on the derived test results, the undetermined coefficients were determined, where $k_{1}$ = 1.69, $k_{2}$ = 0.79, $k_{3}$ = 3.72, and k = 0.17. Finally, the expression for calculating the ultimate shear capacities of the PBL shear connector in SFRCC was proposed.

(12) $$P_{u}=6.76nA_{pr}f_{y}+3.16nA_{cd}\cdot f_{c}\cdot \left(1+0.17V_{f}\frac{L_{f}}{\phi_{f}}\right)+3.72A_{eb}\cdot f_{c}$$

**Figure 16:**
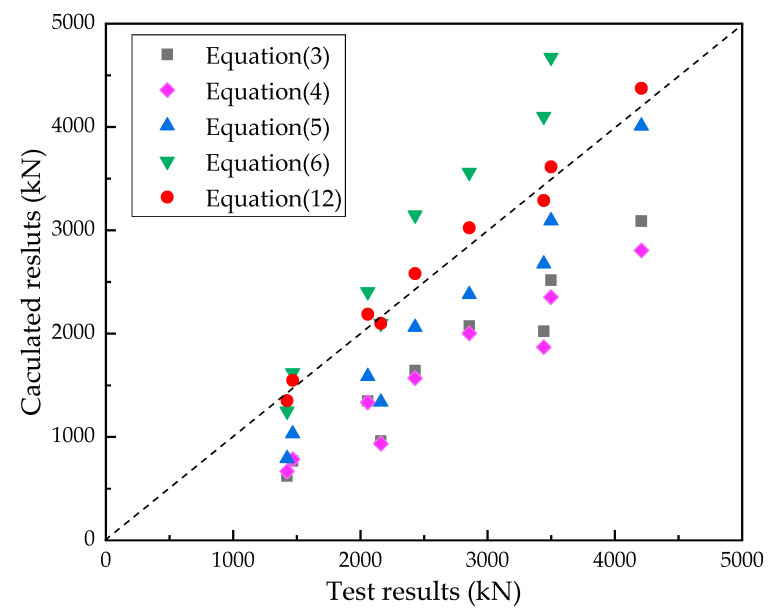
Experimental and numerical ultimate shear capacities of PBL shear connectors in SFRCC.

Figure 16 compares the experimental and numerical ultimate shear-bearing capacities of PBL shear keys in SFRCC. The average value of the ratio of the calculated and test values in Equation (12) was 1.02, indicating that the ultimate shear-bearing capacity formula recommended in this work has high precision, and it would be interesting to prove its effectiveness in other cases.

## 6. Conclusions

The shear performance of PBL shear connectors in steel–NC and steel–SFRCC composite structures was explored by push-out tests and numerical simulations, and based on the experimental and numerical simulation results, an expression for the shear capacity of PBL shear connectors in the steel–SFRCC composite structure was established by considering the bearing actions of concrete dowel, penetrating rebar, and the end part. The main observations of this work are listed below.

(1) The shear bearing capacity, ductility, and shear stiffness of PBL shear connectors in SFRCC were better than those in NC. The ultimate shear bearing capacities of single-hole PBL shear connectors mainly depended on the penetrating reinforcement diameter, whereas that of multi-hole PBL shear connectors depended on both the penetrating reinforcement diameter and the concrete strength.
(2) The failure mode of the single-hole steel–NC specimen was the shear failure of the penetrating reinforcement, and that of the multi-hole steel–NC specimen was concrete cracking. Penetrating rebar shearing and yielding were the main failure modes of the steel–SFRCC specimens, and their steel plate opening failure characteristics were prominent. In comparison with the steel–NC specimens, the steel–SFRCC specimens had slower crack development, smaller crack number and width, and better concrete integrity. However, the single-hole and multi-hole specimens had the problems of excessive concrete strength and the detachment of the perforated steel plates, respectively.
(3) The shear bearing capacity of PBL shear connectors was affected by various factors, such as SFRCC strength, steel plate opening diameter and thickness, and penetrating reinforcement yield strength. Among them, SFRCC strength and penetrating reinforcement yield strength were the main factors, whereas steel plate opening diameter and thickness were secondary factors. Considering the contribution of various factors, an expression for calculating the shear bearing capacities of the PBL shear connectors in the steel–SFRCC composite structure was proposed and had higher precision.

## Figures and Tables

**Table 1:** The mixing ratio of concrete.

Composition	Mixing Quantity (kg/m^3^)	
	NC	SFRCC
Cement	462	672
Water	182	178
Broken stone	1097	993
Sand	587	536
Admixture	4.24	6.88
Silica fume	-	80
Steel fiber (%)	-	144 (2%)

**Table 2:** Mechanical Properties of Materials.

Concrete	Compressive/ Yield Strength (MPa)	Tensile Strength (MPa)	Elastic Modulus (GPa)	Poisson’s Ratio
NC	50.8	1.79	35.9	0.2
SFRCC	112.6	17.2	45.7	0.2
Q345	349	468	206	0.3
HRB400	400	570	200	0.3

**Table 3:** Static performance of each specimen.

Specimens	$P_{u}/\mathrm{kN}$	$P_{Rk}/\mathrm{kN}$	$P_{Rd}/\mathrm{kN}$	$\delta_{u}/\mathrm{mm}$	$\delta_{Rd}/\mathrm{mm}$	D	K/(kN/mm)
N-12-1	1343.99	1209.59	967.67	27.63	0.12	230.25	2687.98
N-12-2	1502.04	1351.84	1081.47	28.83	0.68	42.40	2007.34
N-12-3	1820.44	1638.40	1310.72	29.11	0.48	60.65	2604.92
N-16-1	1327.84	1195.06	956.04	29.55	0.52	56.83	2009.64
N-16-2	1684.31	1515.88	1212.70	20.22	0.58	34.86	2406.50
N-16-3	2306.13	2075.52	1660.41	23.18	0.46	50.39	3442.46
N-20-1	1738.54	1564.69	1251.75	23.70	0.06	395.00	2706.44
N-20-2	2022.33	1820.10	1456.08	24.49	1.67	14.66	2219.60
N-20-3	2852.95	2567.66	2054.12	18.49	0.56	33.02	4005.44
S-12-1	1423.59	1281.23	1024.98	27.80	0.12	231.67	2847.18
S-12-2	2058.09	1852.28	1481.82	31.70	0.39	81.28	3202.50
S-12-3	2856.41	2570.77	2056.62	33.14	0.34	97.47	4803.76
S-16-1	1468.58	1321.72	1057.38	21.10	0.21	100.48	2634.92
S-16-2	2429.57	2186.61	1749.29	29.60	0.73	40.55	3309.42
S-16-3	3498.99	3149.09	2519.27	25.10	0.82	30.61	4399.98
S-20-1	2160.77	1944.69	1555.75	34.50	2.21	15.61	2872.56
S-20-2	3441.31	3097.18	2477.74	34.00	3.76	9.04	3601.26
S-20-3	4208.48	3787.63	3030.11	11.40	0.62	18.39	5821.26

**Table 4:** Typical prediction formulas for the shear capacity of PBL connector.

References	Shear Capacity Equations	
EN 1994-1-1 2004 [36]	$Q_{u}=\left[1.85\left(\pi \left(d_{\rho }{}^{2}-d_{s}^{2}\right)f_{c}/4+\pi d_{s}^{2}f_{st}/4\right)-106.1\times 10^{3}\right]/\gamma$	(3)
JTG D64-201526 [39]	$Q_{u}=1.4\left(d_{\rho }^{2}-d_{s}^{2}\right)f_{cd}+1.2d_{s}^{2}f_{sd}$	(4)
Xiao Lin [40]	$Q_{u}=\beta_{1}\xi \left(t/D\right)A_{cd}f_{c}+\beta_{2}A_{tr}f_{sd}$	(5)
He et al. [23]	$Q_{u}=\left(0.04+0.04V_{f}\frac{L_{f}}{\phi_{f}}\right)A_{b}\sqrt{f_{cu}}+\left(1.06+0.07V_{f}\frac{L_{f}}{\phi_{f}}\right)A_{cd}f_{c}+2.09A_{tr}f_{y}$	(6)

Note: $Q_{u}$ is the shear bearing capacity of the PBL shear connector; $d_{\rho }$ is the opening diameter; $d_{s}$ is the diameter of the penetrating reinforcement; $f_{c}$ is the compressive strength of concrete; $f_{st}$ is the strength of the penetrating reinforcement; γ is the component coefficient; $f_{cd}$ is the design value of concrete strength; $f_{sd}$ is the design value of tensile strength of penetrating reinforcement $\beta_{1}$, $\beta_{2}$ are the influence coefficient of concrete dowels and penetrating reinforcement; ξ is the influence coefficient of stirrup; t is the thickness of the perforated steel plate; D is the opening diameter; $A_{cd}$ is the area of concrete dowels; $A_{tr}$ is the area through the reinforcement; $V_{f}$ is the fiber volume content; $L_{f}$ is the fiber length; and $\phi_{f}$ is the fiber diameter.

**Table 5:** Comparison between test and calculation results.

Specimens	*Q_u_^equ^/Q_u_^Exp^*				
	*Equation (3)*	*Equation (4)*	*Equation (5)*	*Equation (6)*	*Equation (12)*
S-12-1	0.44	0.47	0.56	0.78	0.95
S-12-2	0.65	0.65	0.77	1.37	1.06
S-12-3	0.73	0.70	0.83	1.25	1.06
S-16-1	0.52	0.53	0.70	1.21	1.05
S-16-2	0.68	0.65	0.85	1.30	1.06
S-16-3	0.72	0.67	0.88	1.34	1.03
S-20-1	0.44	0.43	0.62	0.87	0.97
S-20-2	0.59	0.54	0.78	1.39	0.96
S-20-3	0.73	0.67	0.95	1.45	1.04
Average	0.61	0.59	0.77	1.22	1.02

Note: *Q_u_^equ^* is the calculated value, *Q_u_^Exp^* is the test value.

## Data Availability

Not applicable.
